# Effect of gender on the acute effects of whey protein ingestion on energy intake, appetite, gastric emptying and gut hormone responses in healthy young adults

**DOI:** 10.1038/s41387-018-0048-7

**Published:** 2018-07-13

**Authors:** Caroline Giezenaar, Natalie D Luscombe-Marsh, Amy T Hutchison, Kylie Lange, Trygve Hausken, Karen L Jones, Michael Horowitz, Ian Chapman, Stijn Soenen

**Affiliations:** 1 Discipline of Medicine and National Health and Medical Research Council of Australia (NHMRC) Centre of Research Excellence in Translating Nutritional Science to Good Health, The University of Adelaide, Adelaide, SA Australia; 2 CSIRO Animal, Food and Health Sciences, Adelaide, Australia; 3 Department of Medicine, Haukeland University Hospital, Bergen, Norway

## Abstract

**Background/objectives:**

Protein supplements, usually drinks rich in whey protein, are used widely for weight loss purposes in overweight adults. Information comparing the effects of whey protein on appetite and energy intake in men and women is limited. The objective was to compare the acute effects of whey-protein intake on energy intake, appetite, gastric emptying and gut hormones in healthy young men and women.

**Subjects/methods:**

Gastric emptying (3D-ultrasonography), blood glucose and plasma insulin, glucagon, ghrelin, cholecystokinin (CCK), gastric inhibitory polypeptide (GIP) and glucagon-like peptide-1 (GLP-1) concentrations (0–180 min), appetite (visual analogue scales), and ad libitum energy intake from a buffet meal (180–210 min) were determined after ingestion of 30 g (120 kcal) or 70 g (280 kcal) whey protein, or a flavoured-water control drink (~2 kcal) in 8 healthy young men (25 ± 2 y, 72 ± 3 kg, 23 ± 1 kg/m^2^) and 8 women (23 ± 1 y, 64 ± 2 kg, 24 ± 0.4 kg/m^2^).

**Results:**

There was a protein-load effect on gastric emptying, blood glucose, plasma insulin, glucagon, ghrelin, CCK, GIP and GLP-1 concentrations, and perceptions of hunger, desire to eat and prospective food consumption (*P* < 0.05). Ad libitum energy intake (average decrease of 206 ± 39 kcal (15 ± 2%) for men and of 46 ± 54 kcal (0 ± 26%) for women for the mean of the intakes after the 30 and 70 g whey-protein loads) and hunger were suppressed more by whey-protein ingestion in men than women (*P* = 0.046). There was no difference in suppression of energy intake between the 30 and 70 g protein loads (*P* = 0.75, interaction effect *P* = 0.19). Consequently, total energy intake (protein drink plus buffet meal) increased more compared to control in women than men (*P* = 0.010). The drinks emptied more slowly, and plasma glucagon, CCK and GLP-1 increased less after the protein drinks, in women than men (*P* < 0.05).

**Conclusion:**

The acute effects of whey protein ingestion on appetite, energy intake, gastric emptying and gut hormone responses are influenced by gender in healthy young adults.

## Introduction

Supplements and diets high in protein, particularly whey protein, are used frequently for weight loss purposes, in both men and women, based on the rationale that ingestion of protein has a muscle sparing effect and greater satiating effects than carbohydrate and fat^1,2^. Many high-protein diets have been developed and recommended to aid weight loss; well-known versions include the Atkins Diet, South Beach Diet, Zone Diet and Stillman Diet. Our recent studies in healthy young men have shown that whey protein, ingested either orally, or infused intraduodenally, suppresses ad libitum energy intake at a subsequent meal, in excess of the caloric content of the protein load, so that total energy intake (protein plus meal) is less after intake of protein than after a non-caloric control^3,4^. When infused intraduodenally, whey protein increases pyloric and decreases antral and duodenal motility, factors important in the regulation of gastric emptying^3,4^. Oral whey protein ingestion load dependently slows gastric emptying and increases plasma insulin, glucagon, ghrelin, cholecystokinin (CCK), gastric inhibitory polypeptide (GIP) and glucagon-like peptide-1 (GLP-1) concentrations in healthy young men^4^. In younger adults whey empties from the stomach relatively quickly when compared to casein^5^. These effects on gastrointestinal mechanisms are associated with the suppression of appetite and energy intake^3,4^.

It has been reported that after ingestion of liquid and semi-liquid caloric preloads women exhibit lower compensation of energy intake than men, despite comparable perceptions of appetite,^6,7^. For example, in one study of milk or fruit drink preloads, women compensated for the preloads less in their subsequent energy intake than men—on average 50% compensation compared to 107% in men, resulting in an increase in total energy intake (drink plus meal) in women but not men^6^. It has also been reported that after mixed-nutrient meals women have slower gastric emptying^8–11^ and lower plasma glucagon^12^, CCK^13^ and GLP-1 concentrations^12^ than men. It is not known whether gender modulates the acute effects of whey protein, administered in loads representative of a small to large meal (30–70 g, e.g. ~100–250 g serving of lean steak), to suppress energy intake and, if so, what changes in gastrointestinal measures are associated with the suppression of energy intake by protein.

The aim of the study was to compare in healthy young men and women the load-dependent effects of 30 and 70 g whey protein intake on ad libitum energy intake, as well as appetite, gastric emptying, blood glucose and plasma insulin, glucagon, ghrelin, CCK, GIP and GLP-1 concentrations. We hypothesized that women would have less suppression of energy intake, slower gastric emptying and lower gut hormone responses after whey protein ingestion than men.

## Subjects and methods

The methods have been described in detail previously^14^. The study included 8 young men (mean ± SEM: age: 25 ± 2 years; body weight: 72 ± 3 kg; height: 1.79 ± 0.02 m; body mass index (BMI): 23 ± 1 kg/m^2^—the men were included in our previous study relating to energy intake, gastric emptying and perceptions of appetite and gastrointestinal symptoms in healthy older compared to younger men^14^) and 8 young age- (*P* = 0.60) and BMI-matched (*P* = 0.24) women (23 ± 1 years; 64 ± 2 kg; 1.64 ± 0.02 m; 24 ± 0.4 kg/m^2^). Dietary restraint score (Factor 1 of the Three-Factor Eating Questionnaire TFEQ^15^) was not different (*P* *=* 0.65) in men (6 ± 1) and women (7 ± 1) and all subjects were unrestrained eaters (F1 score ≤ 12). Thirteen women were excluded after screening due to low blood iron/ferritin concentrations.

Subjects were studied on three occasions, separated by 3–14 days, to determine the comparative effects of two oral whey protein isolate loads (Fonterra Co-Operative Group Ltd., Palmerston North, New Zealand); 30 g (120 kcal) and 70 g (280 kcal), and a flavoured-water control drink (~2 kcal) in a randomized (www.randomization.com (16 subjects with random permutations)), double-blind, crossover design. In women, study days were scheduled during the follicular phase of their menstrual cycle (i.e., the first 13 days of the cycle) to minimize the potential effect of fluctuations in hormones on gastric emptying and energy intake^16^.

Gastric volume and perceptions of appetite were performed at baseline (during fasting; 0 min) and at 15-min intervals after drink consumption until 180 min^17^. The investigators were blinded during all aspects of the data collection. Gastric volume was measured by three-dimensional (3D) ultrasonography^14^. Gastric retention (%) was calculated as postprandial volume minus fasting volume expressed as percentage of the maximal gastric volume (volume of the drink) during the early (0–60 min) and late (60–180 min) phase of emptying of the drink. Data of gastric retention were imputed by linear interpolation when ultrasound images lacked sufficient clarity. The time at which 50% of the preload drink had emptied from the stomach (50% gastric emptying time; T50; min) and ‘complete’ gastric emptying time (100% gastric emptying time; T100; min), defined as the time when the residual volume of the drink in the stomach was ≤5%, was calculated for all conditions. Complete emptying time was set to 180 min when the residual volume at 180 min was >5%^14^. The overall rate of gastric emptying was calculated as the mean of rates of emptying (kcal/min) during each 15-min interval, respectively, of the early phase (0–60 min), late phase (60 min until complete emptying time per individual) and total time period (0 min until complete emptying time per individual).

Perceptions of appetite and gastrointestinal symptoms were assessed using validated visual analogue scales (VAS)^17^ and blood samples were collected for the measurement of blood glucose and plasma gut hormone concentrations (0–180 min). Blood glucose concentrations (millimoles per liter) were determined immediately after collection by the glucose oxidase method using a portable glucometer (Optium Xceed, Abbott Laboratories, Doncaster, VIC, Australia). Plasma was obtained by centrifugation for 15 min at 3200 rpm at 4 °C and samples were stored at −80 °C for further analysis of hormone concentrations^18^. Plasma total insulin concentrations (milliunits per liter) were determined by enzyme-linked immunosorbent assay (ELISA) immunoassay (10–1113; Mercodia, Uppsala, Sweden). Plasma glucagon (picograms per millilitre), total ghrelin (picograms per millilitre), CCK-8 (picomoles per liter), total GIP (picomoles per liter), total GLP-1 (picomoles per liter) concentrations were determined by radioimmunoassay (RIA)^19–23^. No inhibitors were added^24^. Homoeostatic model assessment (HOMA) index was calculated according to the following formula: insulin concentration at baseline (microunits per liter) × glucose concentration at baseline (nanomoles per liter)/22.5^25^.

At 180 min, subjects were presented, in a room by themselves to limit external distractions, with a standard, cold, buffet meal (including sliced bread, chicken, ham, cheese, margarine, mayonnaise, yoghurt, custard, fruits, fruit salad, orange juice, iced coffee and water^14^) in excess of what they are expected to consume (total energy content of 2457 kcal; 19% protein, 50% carbohydrates, 31% fat) and allowed to eat ad libitum for up to 30 min^26^. Energy intake was calculated both as intake at the buffet meal and as total energy intake, defined as the sum of energy intake at the buffet meal and the energy content of the drink. Absolute change (kcal) and percentage suppression (expressed as % of energy intake of the control day) of energy intake at the buffet meal by a given protein load compared to control were calculated^14^.

The Royal Adelaide Hospital Human Research Ethics Committee approved the study protocol. The study was conducted in accordance with the Declaration of Helsinki and registered as a clinical trial with the Australian New Zealand Clinical Trial Registry (www.anzctr.org.au, registration number ACTRN12611000706976). All participants provided written informed consent prior to their inclusion.

### Data and statistical analyses

On the basis of our previous work^27^, with an observed within-subject standard deviation (SD) of 181 kcal, we estimated an SD using the upper 60% confidence limit of 234 kcal and calculated that eight subjects per group would allow detection of a within-groups (*n* = 8) difference between treatments of 271 kcal and a between groups difference of 353 kcal (*n* = 8 women compared with *n* = 8 men), with power equal to 0.8 and alpha equal to 0.05.

Statistical analyses were performed using SPSS Statistics software (version 21, IBM, Armonk, NY, USA). Effects of gender, protein load and their interaction effect on energy intake and gastric emptying were determined using repeated measures ANOVA, with protein load as the *within-subjec*t factor, and gender as the *between-subject* factor. To adjust for baseline values at each visit as a covariate, a repeated measures mixed effect model, with protein load as the within-subject factor and gender as the between-subject factor was used to test for gender and protein-load effects and their interaction effect on appetite, blood glucose and plasma hormone concentrations. Post hoc comparisons, adjusted for multiple comparisons using Bonferroni’s correction, were performed when there were significant main or interaction effects. *Within-subject* correlations were determined by using a general linear model with fixed slope and random intercept^28^. Areas under the curve (AUC) were calculated using the trapezoidal rule. Assumptions of normality were verified for all outcomes before statistical analysis. Statistical significance was accepted at *P* *<* 0.05. All data are presented as mean values ± SEMs.

## Results

The study protocol was well tolerated by all subjects and there were no untoward effects. All subjects finished eating the test meal in less than 30 min.

### Energy intake

Energy intake at the buffet meal was less in women than men (gender effect *P* = 0.010). On the control day energy intake was 34% lower in women than men (822 ± 109 kcal vs. 1342 ± 131 kcal, *P* *=* 0.010).

**Fig. 1:**
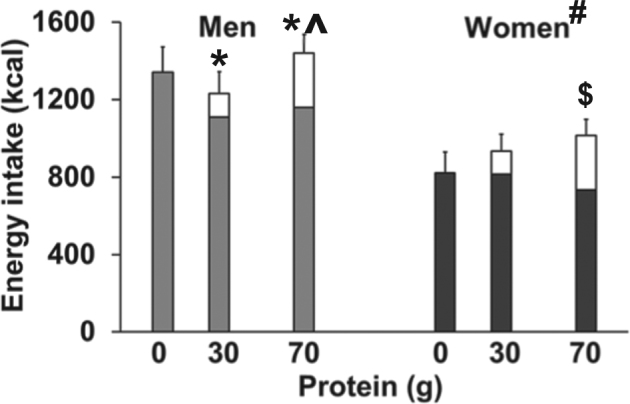
Mean (±SEM) energy intake at the buffet meal (kcal; energy intake in closed bars) in healthy young men (grey shading; *n* = 8) and women (black shading; *n* = 8) after intake of drinks (energy content in open bars) containing flavoured water (control) and whey protein loads of 30 g (120 kcal) and 70 g (280 kcal). Effects of gender and protein-load and interaction effects were determined by using repeated measures ANOVA. Interaction effect of gender by protein-load energy intake at the buffet meal *P* = 0.046 and total energy intake (drink + buffet meal) *P* *=* 0.046. ^#^ Effect of gender: energy intake *P* = 0.010 and total energy intake *P* = 0.010 were higher in men than women. Effect of protein load: energy intake *P* = 0.008 and total energy intake *P* *=* 0.002. * Post hoc effects: energy intake was lower after the 30 g (*P* *=* 0.001) and 70 g (*P* *=* 0.049) protein drink compared to control in men. ^^^ Post hoc effects: total energy intake was higher after the 70 g compared to the 30 g protein drink in men (*P* *=* 0.021); ^$^ Post hoc effects: total energy was higher after the 70 g protein drink compared to control in women (*P* *=* 0.033)

Energy intake at the buffet meal was suppressed more after the protein loads compared to control in men than women, with no suppression in women (protein-load effect *P* = 0.008, interaction effect of gender by protein-load *P* = 0.046; Fig. 1). The mean suppression of the 30 and 70 g protein loads compared to control was 206 ± 39 kcal or 15 ± 2% for men, while it was 46 ± 54 kcal or 0 ± 26% for women (gender effect *P* = 0.032). There was no difference in suppression between the 30 and 70 g protein loads (protein-load effect *P* = 0.75, interaction effect *P* = 0.19).

There was a protein-load effect (*P* = 0.002) on total energy intake (drink plus buffet meal), which was higher in women than men (gender effect *P* = 0.010, interaction effect *P* = 0.046), Compared to the total energy intake on the control day, total energy intake on the 30 and 70 g protein days increased 22 ± 13 and 35 ± 15%, respectively, in women, and decreased 8 ± 3% and increase 10 ± 5%, respectively, in men. Total energy intake was higher after the 70 g compared to the 30 g protein load in men (*P* *=* 0.021), and after the 70 g protein drink compared to control in women (*P* *=* 0.033).

### Macronutrient intake at the buffet meal

At the buffet meal, compared to men, women consumed a higher percentage of their energy intake as protein (average of all three study days: women: 24 ± 1%, men: 20 ± 1%; gender effect *P* *=* 0.023, protein-load effect *P* *=* 0.31, interaction effect, *P* *=* 0.60) and fat (women: 36 ± 1%, men: 28 ± 1%; gender effect *P* *=* 0.006, protein-load effect *P* *=* 0.09, interaction effect *P* *=* 0.85), and less as carbohydrate (women: 41 ± 3%, men: 52 ± 2%; gender effect *P* *=* 0.001, protein-load effect *P* *=* 0.13, interaction effect *P* *=* 0.98).

### Appetite

Baseline hunger, desire to eat, prospective food consumption, fullness, nausea and bloating were comparable in men and women (all *P* > 0.05). Protein drink ingestion was associated with a load-dependent decrease in perceptions (AUC and ratings immediately before the buffet meal at 180 min) of hunger (*P* *=* 0.002 and *P* *=* 0.002), desire to eat (*P* *=* 0.001 and *P* *<* 0.001) and prospective food consumption (*P* *=* 0.001 and *P* *=* 0.005).

**Fig. 2:**
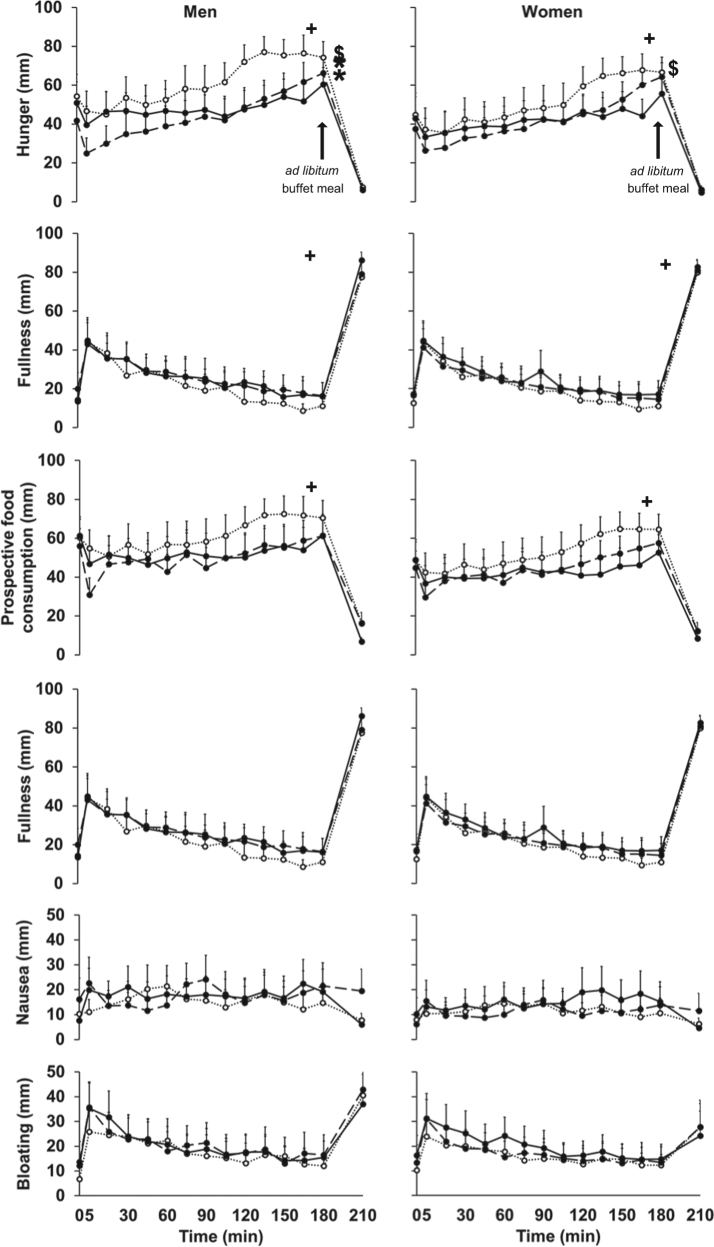
Mean (± SEM) Visual analogue score (VAS, mm) of hunger, desire to eat, prospective food consumption, fullness, nausea and bloating in healthy young men (*n* = 8) and women (*n* = 8) after drinks containing flavoured water (control; dotted line with open circles) and whey protein loads of 30 g (dashed line with closed circles) or 70 g (solid line with closed circles). Effects of gender and protein-load and interaction effects were determined by using repeated measures ANOVA including baseline values at each treatment visit as a covariate. ^*+*^ *P* < 0.005 Effect of protein load: perceptions (area under the curve; AUC) of hunger (*P* *=* 0.002), desire to eat (*P* *=* 0.001) and prospective food consumption (*P* *=* 0.001) protein-load dependently increased after drink ingestion. ^$^ *P* *=* 0.0016 Interaction effect of gender by protein-load: perceptions of hunger were lower in women than men after the control drink. * *P* *<* 0.005 Interaction effect of gender by protein-load: in men hunger was suppressed after both 30 g (Post hoc *P* = 0.004) and 70 g (Post hoc *P* *<* 0.001) protein loads compared to control

Hunger ratings were lower in women than men during the control day, and decreased in men, but not women, after both 30 g (*P* = 0.004) and 70 g (*P* *<* 0.001) protein loads compared to control day values (gender effect *P* *=* 0.08, interaction effect of gender by protein-load *P* *=* 0.014; Fig. 2).

### Gastric emptying

**Fig. 3:**
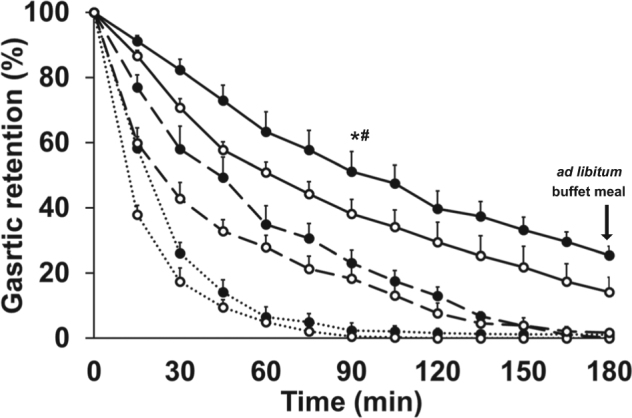
Mean (±SEM) gastric retention (%) in healthy young men (*n* = 8; open circles) and women (*n* = 8; closed circles) after drinks containing flavoured water (control; dotted line) and whey protein loads of 30 g (dashed line) or 70 g (solid line). Effects of gender and protein-load and interaction effects were determined by using repeated measures ANOVA. * *P* < 0.05 50% gastric emptying time (T50): effect of gender and protein-load (interaction effect of gender by protein-load *P* = 0.17). ^#^ *P* < 0.05 area under the curve (AUC): effect of gender and protein-load (interaction effect of gender by protein-load *P* = 0.34)

Gastric emptying parameters are detailed in Table 1. Baseline gastric volumes were comparable in men (31 ± 6 mL) and women (34 ± 4 mL, *P* = 0.69) and between study days (*P* = 0.41). The control (water) and the 30 g protein drinks emptied in an overall non-linear pattern, whereas the pattern of the 70 g protein drink was linear (Fig. 3). Gastric retention (AUC % decrease in stomach volume compared to directly after drink ingestion, *P* < 0.001), gastric emptying halftime (T50, *P* *<* 0.001), complete emptying time (T100, *P* *<* 0.001) and the rate of gastric emptying (kcal/min, *P* *<* 0.001) protein-load dependently increased after drink ingestion. The drinks emptied slower in women than men; gastric retentions were higher in women compared to men (gender effect *P* = 0.021, interaction effect of gender by protein-load *P* = 0.34).

**Table 1:** Gastric emptying parameters of whey protein (30 and 70 g) and control drinks in healthy young men and womens

	Men (*n* = 8)			Women (*n* = 8)		
	0 g	30 g (120 kcal)	70 g (280 kcal)	0 g	30 g (120 kcal)	70 g (280 kcal)
50% emptying time (T50; min) ^+^	12 ± 1	25 ± 4	72 ± 13	19 ± 1	39 ± 5	98 ± 14
100% emptying time (100, min) ^+^	60 ± 7	126 ± 14	171 ± 6	81 ± 15	176 ± 4	180 ± 0
Rate of gastric emptying (kcal/min) ^1,**+**^		1.0 ± 0.1	1.5 ± 0.1		0.9 ± 0.0	1.2 ± 0.0
Early phase rate of gastric emptying ^1 +^		1.4 ± 0.1	2.3 ± 0.1		1.3 ± 0.1	1.7 ± 0.3
Late phase rate of gastric emptying ^1 +^		0.3 ± 0.1	1.0 ± 0.2		0.5 ± 0.1	1.0 ± 0.1
Amount emptied at 60 min (%) ^+^	95 ± 1	72 ± 4	49 ± 3	93 ± 3	65 ± 6	37 ± 6
Amount emptied at 180 min (%) ^**+**,^*	100 ± 0^a^	98 ± 1^a^	86 ± 5^b^	99 ± 1^a^	100 ± 0^a^	75 ± 3^b^

All values are mean ± SEM. Effects of gender and protein-load and interaction effects were determined by repeated measures ANOVA

^1^Rate of gastric emptying was calculated as mean of rates of emptying during each 15-min interval, respectively, of the early phase (i.e. 0–60 min), the late phase (i.e. 60 min until 100% emptying time per individual) and total time period (i.e. 0 min until 100% emptying time per individual). ^**+**^ *P* < 0.001 Effect of protein load. *P* ≤ 0.1. *P < 0.005 Interaction effect of gender by protein-load: amount emptied at 180 min

^a,b^*P* *<* 0.05, post hoc test: different letter indicates significant difference between drink conditions.

### Blood glucose and plasma gut hormone concentrations

Baseline concentrations of blood glucose (5.4 ± 0.1 mmol/L) and plasma insulin (5.3 ± 0.6 mU/L), glucagon (68 ± 4 pg/mL), ghrelin (1507 ± 207 pg/mL), CCK (3.3 ± 0.4 pmol/L) and GIP (16 ± 2 pmol/L), and HOMA index (1.3 ± 0.1) were comparable in men and women (*P* > 0.05), while plasma GLP-1 concentrations were lower in women (16.5 ± 0.9 pmol/L) than men (20.6 ± 2.0 pmol/L, *P* *<* 0.001).

**Fig. 4:**
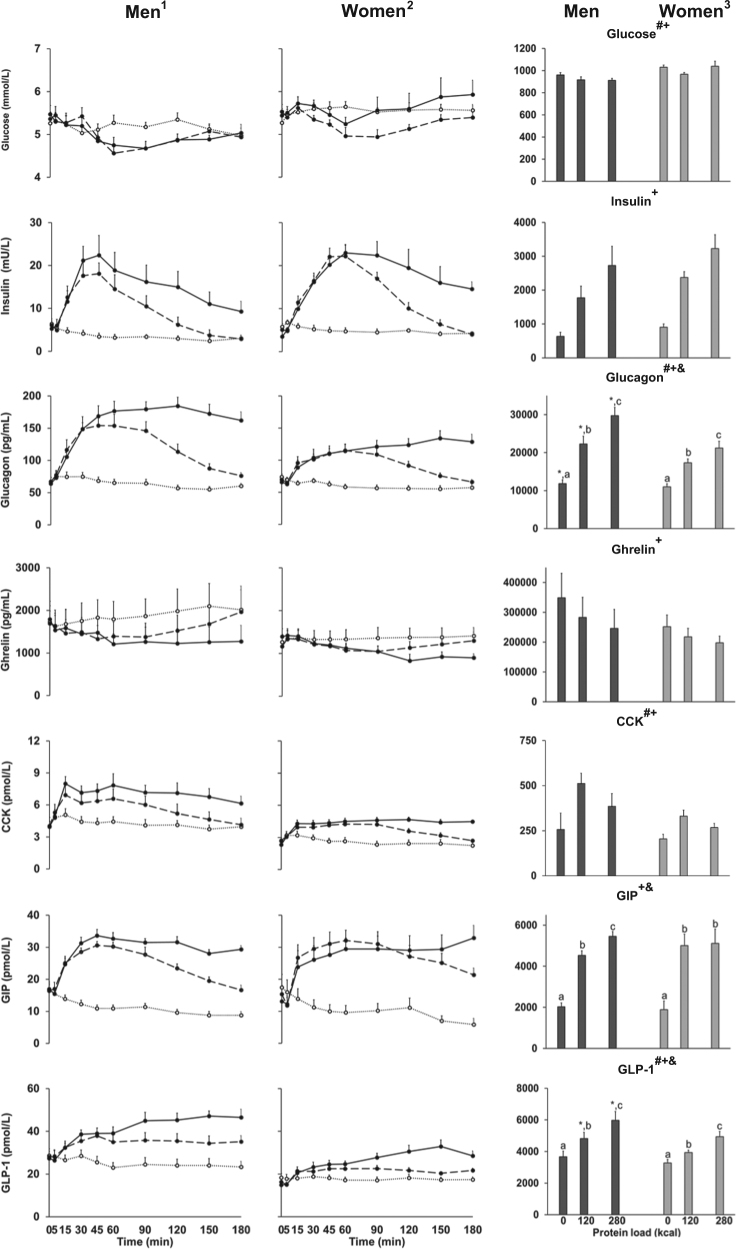
^1,2^Mean (±SEM) and ^3^ area under the curve (AUC) blood glucose and plasma insulin, glucagon, ghrelin, cholecystokinin (CCK), gastric inhibitory polypeptide (GIP) and glucagon-like peptide-1 (GLP-1) concentrations in healthy young men (*n* = 8, *n* = 7 for GIP and GLP-1) and women (*n* = 8) after drinks containing flavoured water (control; dotted line with open circles) and whey protein loads of 30 g (dashed line with closed circles) or 70 g (solid line with closed circles). Effects of gender and protein-load and interaction effects of the AUC were determined by using repeated measures ANOVA including baseline values at each treatment visit as a covariate and post hoc Bonferroni corrections. ^#^ *P* < 0.05 Effect of gender; ^+^ *P* < 0.001 Effect of protein load; ^&^ *P* < 0.05 Interaction effect of gender by protein-load; * *P* *<* 0.05 Interaction effect post hoc test: men compared to women (AUC); ^a, b, c^ *P* *<* 0.05 Interaction effect post hoc test: a different letter indicates a difference between protein loads within gender group (AUC)

AUC blood glucose and plasma ghrelin decreased, and plasma insulin, glucagon, CCK, GIP, GLP-1 concentrations increased in a load-dependent fashion after the protein preloads (all *P* < 0.01; Fig. 4). 60 and 180 min plasma ghrelin concentrations decreased and plasma insulin, glucagon, CCK, GIP and GLP-1 concentrations increased in a protein-load dependent fashion (all *P* *<* 0.05; Table 2). AUC blood glucose concentrations were lower after the 30 g protein drink compared to control. 60-min plasma ghrelin concentrations were lower after both protein drinks compared to control (all *P* *<* 0.05). AUC plasma ghrelin concentrations were lower after the 70 g protein drink compared to the 30 g protein and control drinks. 60-min and AUC plasma insulin, glucagon, CCK, GIP and GLP-1 concentrations were higher after both protein drinks compared to control, and 180-min and AUC concentrations after the 70 g compared to the 30 g protein drink (all *P* *<* 0.05). 60-min plasma GLP-1 concentrations were higher after 70 g compared to 30 g protein (*P* *=* 0.036).

**Table 2:** Concentrations of glucose, insulin, glucagon, ghrelin, cholecystokinin (CCK), gastric inhibitory polypeptide (GIP) and glucagon-like peptide-1 (GLP-1)

A	Gender effect			B	Protein-load effect			
	Men	Women	Average		Control	30 g protein	70 g protein	Average
*60* *min*								
Glucose	4.9 ± 0.1	5.3 ± 0.1			5.5 ± 0.1^a^	4.8 ± 0.2^b^	5.0 ± 0.1^b^	
Insulin			14.4 ± 1.5		4.0 ± 0.5^a^	18.3 ± 2.3^b^	20.9 ± 2.3^b^	
Glucagon	132 ± 12	96 ± 6			62 ± 3^a^	134 ± 11^b^	145 ± 12^b^	
Ghrelin			1319 ± 180		1563 ± 236^a^	1230 ± 179^b^	1165 ± 130^b^	
CCK	6.3 ± 0.7	3.8 ± 0.3			3.5 ± 0.4^a^	5.4 ± 0.6^b^	6.2 ± 0.7^b^	
GIP			24.2 ± 1.3		10 ± 1^a^	31 ± 2^b^	31 ± 2^b^	
GLP-1	32.4 ± 3.1	21.4 ± 1.2			20 ± 2^a^	29 ± 3^b^	32 ± 2^c^	
*180* *min*								
Glucose	5.0 ± 0.1	5.6 ± 0.2						5.3 ± 0.1
Insulin	5.1 ± 1.2	7.6 ± 0.7			3.6 ± 0.4^a^	3.5 ± 0.4^a^	11.9 ± 1.6^b^	
Glucagon	99 ± 6	84 ± 6			59 ± 3^a^	71 ± 7^b^	145 ± 10^c^	
Ghrelin			1477 ± 253		1713 ± 294^a^	1633 ± 281^a^	1085 ± 194^b^	
CCK	4.8 ± 0.5	3.1 ± 0.2			3.1 ± 0.3^a^	3.4 ± 0.4^a^	5.3 ± 0.4^b^	
GIP			19.2 ± 1.2		7 ± 1^a^	19 ± 1^b^	31 ± 2^c^	
GLP-1	35 ± 3	23 ± 1			20 ± 2^a^	28 ± 2^b^	38 ± 3^c^	

C	Interaction effect					
	Men			Women		
	Control	30 g protein	70 g protein	Control	30 g protein	70 g protein
*60* *min*						
Glucagon	65 ± 5*^,a^	154 ± 18*^,b^	177 ± 15*^,c^	58 ± 4^a^	115 ± 10^b^	114 ± 11^b^
GLP-1	23.0 ± 2.4^a^	35.0 ± 4.2^b^	39.1 ± 3.0*^,b^	17.2 ± 1.3^a^	22.5 ± 1.4^,b^	24.7 ± 1.4^b^
*180* *min*						
GIP	7.6 ± 1.7*^,a^	19.6 ± 1.8*^,b^	31.8 ± 3.7^c^	5.9 ± 1.9^a^	21.4 ± 2.2^b^	32.9 ± 3.9^c^

Main gender (A) and protein-load effects (B), and interaction effects (C) of gender by protein-load for mean (± SEM) 60-min and 180-min concentrations of blood glucose (mmol/L) and plasma insulin (mU/L), glucagon (pg/mL), ghrelin (pg/mL), cholecystokinin (CCK; pmol/L), gastric inhibitory polypeptide (GIP; pmol/L) and glucagon-like peptide-1 (GLP-1; pmol/L) in healthy young men (*n* = 8, *n* = 7 for GIP and GLP-1) and women (*n* = 8) after control (~2 kcal) and 30 g (120 kcal) or 70 g (280 kcal) protein loads. Results are presented separately for gender or protein-load if the main effect was significant, in case of non-significance the average was presented. Interaction effects of gender by protein-load are given if the effect was significant. Effects of gender and protein-load and interaction effects were determined by using repeated measures ANOVA including baseline values at each treatment visit as a covariate and post hoc Bonferroni corrections. Post hoc effects: * *P* < 0.05: men vs. women; a,b,c *P* < 0.05: a different letter indicates a significant difference between drink-conditions within subject group.

Women compared to men had higher 60-min and AUC blood glucose concentrations (*P* < 0.05), and lower 60-min, 180-min and AUC plasma glucagon, CCK and GLP-1 concentrations (all *P* < 0.05). Women compared to men had higher 60-min and AUC plasma glucagon concentrations, lower 60-min and AUC plasma GLP-1 and 180-min and AUC GIP concentrations (gender by protein-load interactions all *P* *<* 0.05).

### Relationships between energy intake, appetite, gastric emptying and gut hormones

Energy intake was, *within subjects*, inversely related to 180-min plasma insulin, (*r* = −0.37, *P* *=* 0.032), CCK (r = −0.36, *P* *=* 0.041), GIP (r = −0.37, *P* *=* 0.033) and GLP-1 (r = −0.37, *P* *=* 0.001) concentrations, and positively related to perceptions of hunger (r = 0.37, *P* *=* 0.032), desire to eat (r = −0.53, *P* *=* 0.002) and prospective food consumption (r = 0.40, *P* *=* 0.022). GIP concentrations were related to GLP-1 concentrations (r = 0.78 *P* < 0.001), while ghrelin concentrations were inversely related to insulin concentrations (*r* = −0.63 *P* < 0.001).

## Discussion

This study examined the acute effects of oral whey protein ingestion on energy intake, perceptions of appetite and gastrointestinal symptoms, gastric emptying, blood glucose and plasma gut hormone concentrations in women and men. This is the first study we are aware of to compare the effect of gender on these parameters after pure protein intake.

There was a load-dependent suppressive effect of protein on perceptions of hunger, desire to eat, prospective food consumption, and blood glucose and plasma ghrelin concentrations, slowing of gastric emptying, and increase of plasma insulin, glucagon, CCK, GIP and GLP-1 concentrations. Hunger and energy intake were less in women than men. The protein drinks emptied from the stomach more slowly and plasma glucagon, CCK and GLP-1 concentrations increased less after protein in women than men. The major finding was that hunger and ad libitum energy intake were suppressed by the whey protein ingestion in men, but not in women. Men had a 15% reduction in ad libitum food intake at the buffet meal after the protein drinks, whereas there was no suppression in women. The suppression in men resulted in almost 100% compensation for the energy content of the protein drinks (206 kcal reduction vs. 200 kcal mean energy content of the two protein drinks), whereas there was no compensation in women. Consequently, compared to the control day, total energy intake (protein drink *plus* buffet test meal) was increased by ingestion of the protein drinks in women (∼150 kcal (∼30%)), with no effect in men.

There is evidence that protein has greater satiating effects than the other macronutrients (carbohydrate and fat^29–32^) and that enhanced protein diets can facilitate weight loss^2,33^; protein diets are widely used for this purpose by both men and women trying to lose weight. There is also evidence, however, that men lose weight more easily than women on energy-restricted diets^34^, and that women, when compared to men compensate less for energy intake after mixed macronutrient drinks^6^ and semi-liquid (yoghurt) preloads^7^. This may be due, at least in part, to the lower satiating effect of protein in young women than men, demonstrated for the first time in the present study. The outcomes of this study may therefore have important implications for the types of dietary modifications recommended to promote weight loss in those trying to lose weight. Less emphasis on protein enrichment for women may be appropriate.

Appetite and energy intake are dependent on the precise co-ordination of interrelated gastric and small intestinal mechanisms, triggered by the interaction of these organs with ingested nutrients. The rate of gastric emptying has an important role in mediating gut hormone release in response to protein, fat and carbohydrates^3,35–37^, and emptying of food content from the stomach itself is slowed by feedback mechanisms originating in the small intestine, including the release of CCK and GLP-1^38,39^. As expected, gastric emptying was markedly and dose-dependently slowed by whey protein ingestion in this study, with the 50% gastric emptying time more than doubling compared to the control day on the 30 g protein day, and doubling again from the 30 to 70 g protein day. Consequently gastric emptying of protein into the small intestine was completed earlier after the 30 g than the 70 g whey loads, which probably accounts for the earlier return to baseline after the 30 vs. 70 g protein loads of plasma concentrations of insulin, glucagon, ghrelin and CCK in both men and women. After protein ingestion there was an early increase in plasma concentrations of CCK and GIP, both mainly produced in the duodenum and proximal jejunum, reaching a plateau from 15–30 min onwards, while concentrations of GLP-1, produced more distally in the ileum, showed a more constant increase. Rates of gastric emptying of the protein drinks were at the lower end of the published normal range (1–4 kcal/min 24, 41–43), with faster gastric emptying rates during the 70 than 30 g protein loads. Our finding that gastric emptying was slower in women than men is consistent with the results of most^8–11^, but not all, previous studies^11,40^.

The finding of lower glucagon, CCK and GLP-1 concentrations after protein ingestion in women than men is consistent with previous reports that women have lower plasma concentrations of glucagon, CCK and GLP-1 than men after mixed-nutrient liquid intake (, but not after glucose^41^, or corn oil^42^, suggesting the gender difference in responses to mixed-nutrient intakes relate to different responses to protein, not carbohydrate or fat. Both CCK and GLP-1 suppress appetite and food intake^43^, so the reduced increases in circulating concentrations of these hormones after protein ingestion in women than men, possibly at least in part due to the associated slower gastric emptying of protein in women than men—provide one possible explanation for the observed, reduced satiating effect of whey protein in women than men. In the present study plasma insulin, ghrelin and GIP concentrations were comparable in men and women, consistent with most previous reports; for insulin after oral glucose^41,44^, insulin and ghrelin after mixed-nutrient ingestion^12^, and insulin and GIP during intravenous glucose administration^44^. Ghrelin concentrations have, however, also been reported to be higher in women than men after oral loads of glucose and lipids^45^.

We do not know why healthy, young women and men respond differently to protein ingestion. It has been suggested that sex hormones affect food intake^46^. Pre-menopausal women are reported to have slower gastric emptying and lower appetite, food intake and plasma GLP-1 concentrations during the follicular than luteal phase, without changes in CCK concentrations^16^. The young adult, pre-menopausal women in the present study were investigated during the follicular phase of the menstrual cycle, so it is possible that some of the differences between women and men observed, including reduced suppression of appetite and food intake by protein, would have been reduced or absent if the women were examined during the luteal phase of their cycles.

Energy intake at the buffet meal was assessed 3 h after drink ingestion, to allow for complete emptying of the drinks from the stomach and thus detailed assessment of gastric emptying. Consequently the buffet meal was presented to subject when their stomach was (nearly) empty in both women and men. Energy intake at the buffet meal was related to perceptions of appetite and plasma gut hormone concentrations immediately before the meal. Energy intake and appetite were also related to the rate of emptying of the whey protein drink from the stomach and plasma gut hormone responses, which were interrelated; the greater the increase in plasma insulin, glucagon, CCK, GIP and GLP-1 and decrease in ghrelin concentrations, the slower the drink emptied from the stomach within a subject—70 < 30 < 0 g—the lower the perceptions of appetite, and the lower the subsequent energy intake at the buffet meal.

Women tend to restrain their food intake more than men, potentially caused by social pressure to achieve an ideal body shape, and show more signs of disinhibition of restrained eating^47^. In this study men and women were not dietary restraint, as assessed by the TFEQ^15^, and restraint score was not significantly different between men and women and thus did not explain the difference in suppression of food intake by whey protein ingestion in men and women.

Our study has several limitations, including the relatively small number of subjects. Nevertheless, the results appear clear-cut. The protein preload drinks were selected to be iso-caloric for both men and women. Women, on average, have lower energy requirements than men, so the drinks given to women in this study could be considered to be larger than those given to the men relative to energy requirements. This, if anything would be expected to lead to greater suppression of appetite and food intake in women than men, the opposite of what we found. As mentioned above, we do not know if these findings in women studied during the follicular phase of their menstrual cycle also extend to women in the luteal phase or on hormonal medications such as the oral contraceptive pill. While the drinks were matched for taste, we did not assess the subject’s perceptions of taste, pleasantness and/or palatability of the drinks. Blood glucose was measured by a glucometer, which is less than optimal, however, the results appear to be clear-cut. Blood glucose and plasma gut hormone concentrations were determined in response to ingestion of protein and control drink but not during or after the buffet meal.

In summary, in young healthy women, when compared to men, whey protein drinks emptied slower from the stomach, increases in plasma glucagon, CCK and GLP-1 concentrations after protein were reduced, and there was less suppression of energy intake and hunger—in fact none in women at these protein doses. These findings have potential implications for the efficacy of ingesting whey or other proteins to decrease overall food intake and achieve voluntary weight loss in women. Further studies are needed to determine how broadly these findings apply to other settings, including the use of other proteins, while longer-term studies will be needed to determine the effects of ingesting whey or other proteins on chronic changes in food intake, body weight and body composition.
